# Comparison of the Content of Extractives in the Bark of the Trunk and the Bark of the Branches of Silver Fir (*Abies alba* Mill.)

**DOI:** 10.3390/molecules28010225

**Published:** 2022-12-27

**Authors:** Viljem Vek, Tjaša Šmidovnik, Miha Humar, Ida Poljanšek, Primož Oven

**Affiliations:** 1 Department of Wood Science and Technology, Biotechnical Faculty, University of Ljubljana, Jamnikarjeva 101, SI-1000 Ljubljana, Slovenia; 2 Department of Forestry, Biotechnical Faculty, University of Ljubljana, Jamnikarjeva 101, SI-1000 Ljubljana, Slovenia

**Keywords:** bark, *Abies alba*, chromatography, antioxidant properties, antifungal properties

## Abstract

The main objective of our study was to investigate the possible differences in the chemical composition of extractives from the bark of silver fir (*Abies alba*) with respect to the location of the bark sample on the tree, viz. differences in extract composition between stem bark and branch bark samples. Extractives in the bark samples from branches, depending on the distance of the sample from the trunk, were also analysed, and the stem bark samples were analysed with respect to their inner and outer parts. The results of the chemical analysis of extractives were supported by information about their antifungal and antioxidant effects. After felling and sampling silver fir trees, the collected bark samples were ground and freeze-dried. Extraction of bark samples was followed by a system of accelerated extraction using only water as a solvent. The extracts were analysed chemically using gravimetry, spectrophotometry and chromatography. Free-radical-scavenging activity was measured using the DPPH method, and the antifungal effect towards three moulds and three wood-decaying fungi was investigated with antifungal assay using the agar well diffusion method. It was found that the moisture content in bark samples decreased intensively just after the bark samples were peeled off the stem. Detailed chromatographic analysis showed that the bark extracts contained 14 compounds, among which phenolic acids, flavonoids and lignans were found to be the characteristic ones. The content of hydrophilic extractives in the branch bark samples decreased with increasing distance of the sample location from the tree stem. The largest amounts of phenolic extractives were measured in stem bark, followed by branch bark sampled at the point at which the branch entered the tree. Analysis of the separated parts of the bark showed that the outer layers of stem bark contained larger amounts of phenolic extractives, as well catechin and epicatechin, compared to the inner layers. Concentrated extracts of branch bark showed the largest free-radical-scavenging activity among the investigated samples, while strong antifungal effects of the bark extract were not found.

## 1. Introduction

With the fast development of biorefinery concepts, extensive research is being devoted to the valorisation potential of versatile plant material. Forest trees remain an irreplaceable source of wood for traditional purposes, such as construction, furniture and musical instruments, to mention only a few; however, they are also receiving increased attention as biorefinery raw material. One of the species with presumably underestimated potential in Europe is silver fir (*Abies alba* Mill.). This species grows in the mountain vegetation belt in Europe, from the Pyrenees to Normandy, east to the Alps and the Carpathians, and south to Italy, Bulgaria and Greece, usually at relatively high altitudes between 500 and 2000 m.a.s.l., but it can be also found in the lowlands of France, Poland and Ukraine [1].

Bark is the second-most valuable product from forestry after wood, comprising 10% to 15% of the stem volume. Bark is considered to be waste or residue in the wood industry and is often used only as an energy source. The morphological and chemical composition of bark is heterogeneous and variable, not only between tree species, but also within an individual tree, mostly due to age-related alterations of these tissues [2]. In older parts of most tree species, including silver fir, bark is composed of inner/living and outer/dead bark (rhytidome), which are separated by the youngest and hence deepest periderm [3]. Bark in younger parts of trees is without the rhytidome and is covered by a superficial periderm [4]. Bark is the non-technical term describing all tissues outside the vascular cambium [5]. It fulfils important physiological functions in a living tree: translocation and storage of photosynthetic products [6] as well as protection and defence against biotic and abiotic agents [7,8]. This is reflected in the chemistry of bark, which differs from wood with respect to composition and the amounts and ratios of structural (cellulose, hemicelluloses, lignin, suberin) and non-structural compounds (extractives) [9,10,11]. The bark of conifers can contain up to six times more extractives compared to stemwood, as reported by Routa et al. [11]. Extractable non-structural compounds that are present in the bark of silver fir are soluble carbohydrates, terpenes, aliphatic alcohols and fatty acids and polyphenols, such as stilbens, flavonoids, lignans and tanins [12,13,14,15,16,17,18,19]. 

Based on a review of the aforementioned literature, we initiated this preliminary study to examine the additional sources of variability in the content of bark extractives in silver fir. Silver fir bark extract from the trunk is already used commercially as a dietary supplement. Our study showed that an extract of silver fir bark from branches, with a slightly different composition, could be marketed for the same or a different purpose, since this extract has good antioxidant activity and excellent fungicidal potential.

The main goal of the study was a qualitative and quantitative examination of silver fir (*Abies alba* Mill.) bark water extract. We analysed the content of hydrophilic extracts, total phenols and individual polyphenols using HPLC. We analysed how the content of extracts differed according to the location of the bark. Differences between the content of extractives in branch bark and trunk bark were analysed. In addition, differences according to the distance of the branch bark sample from the stem surface were investigated. Another analysis included differences in the chemical composition of the inner and outer part of the bark. Finally, the antioxidant and fungicidal potential of silver fir bark extract (*Abies alba* Mill.) was studied. 

## 2. Results

The average values of bark moisture content as a function of time are presented in Table 1. The moisture of the samples decreased with time and stabilised at 35% moisture after 50 days, when it reached relative humidity.

Our samples were stored on an asphalt surface under a roof, so humidity had no direct effect on the moisture content. It was only influenced by the relative humidity.

**Figure 1:**
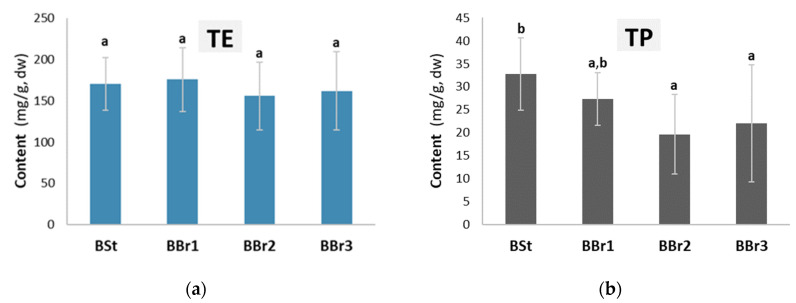
Contents of water-soluble extractives (TE; (**a**)) and total phenols (TP; (**b**)) in the stem and branch bark samples of silver fir (*Abies alba*). Results are expressed in milligrams of extracted compounds per gram of dried bark (mg/g, dw). The different letters a and b at the top of the error bars indicate statistically significant differences at a 95% confidence level (LSD test).

Chemical analysis revealed that the stem and branch bark samples of silver fir (*Abies alba*) contained an average of 16.7% (*w*/*w*, dw) of water-soluble extractives and 2.67% (*w*/*w*, dw) of total phenols (Figure 1). Differences in the content of total extractives were not significant among the investigated samples (ANOVA, *p* = 0.651), while the spectrophotometric method showed the highest amounts of total phenols in stem bark (BSt) and in the bark at the base (BBr1) of a branch (LSD test, ANOVA, *p* < 0.051; Figure 1). It can be concluded from Figure 1 that the average total phenolic content in the hydrophilic extracts is about 15%.

**Figure 2:**
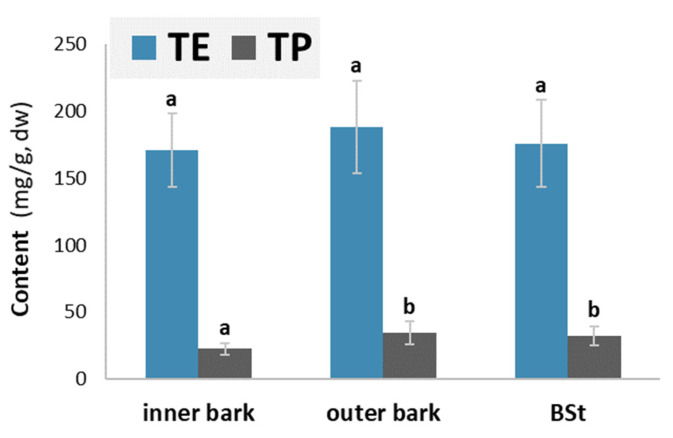
Contents of water-soluble extractives (TE) and total phenols (TP) in inner bark samples, outer bark samples and stem bark samples of silver fir (*Abies alba*). Results are expressed in milligrams of extracted compounds per gram of dried bark (mg/g, dw). The different letters a and b at the top of the error bars indicate statistically significant differences at a 95% confidence level (LSD test).

Figure 2 provides information about the average content of water-soluble extractives (TE) and total phenols (TP) in the samples of the inner bark, outer bark and stem bark of silver fir (*Abies alba*). 

The average content of hydrophilic extractives was comparable in the inner and outer bark layers (ANOVA, *p* = 0.6170). The content of total phenolics was lower in the inner layer than in the outer part of the bark. The outer layer of the bark contained on average 17.3 mg/g more hydrophilic extracts and 14.87 mg/g more phenols than the inner layer of the bark. The higher content of hydrophilic extracts and total phenols may be attributed to the anatomical differences between the inner and outer bark layers. Secondary changes begin in the outer, older part of the living bark. This means the death of parenchyma cells and an accumulation of phenolic substances in their lumen. The phenols present in the cork cells of the periderm probably also contribute to the higher content.

Thin-layer chromatography showed that our samples contained (at least) 14 compounds. We identified some of them using standard mixtures. To determine all compounds, we relied on the literature [20]. Pinoresinol, matairesinol, hydroxymatairesinol, lariciresinol, secoisolariciresinol, lignan A and oligolingnans were identified using TLC. The TLC plate in Table 2 shows that large molecules and polar compounds remained on the baseline.

**Figure 3:**
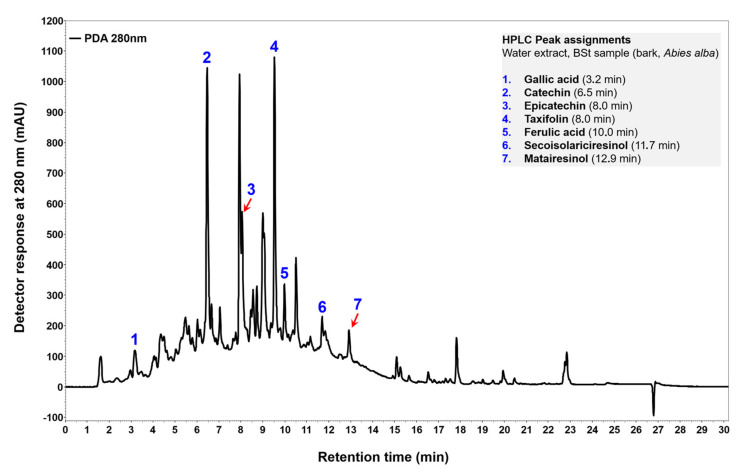
HPLC–PDA chromatogram of silver fir (*Abies alba*) water extract monitored at 280 nm. Sample of stem bark (BSt). The chromatogram is equipped with the list of separated phenolic compounds.

**Figure 4:**
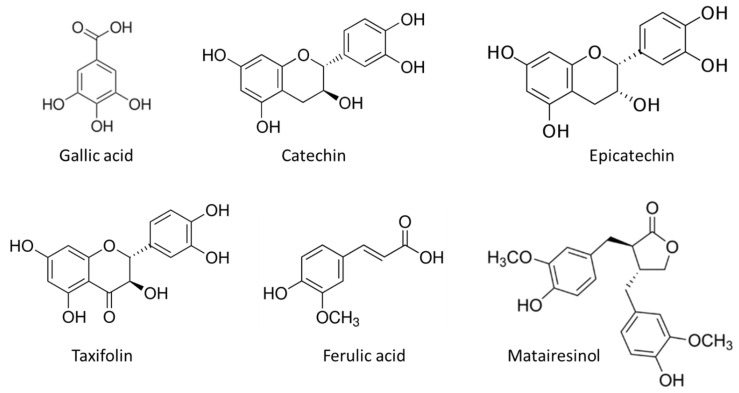
Chemical structure of the most abundant compounds in the stem bark sample of silver fir: catechin, epicatechin, taxifolin, ferulic acid and matairesinol.

**Figure 5:**
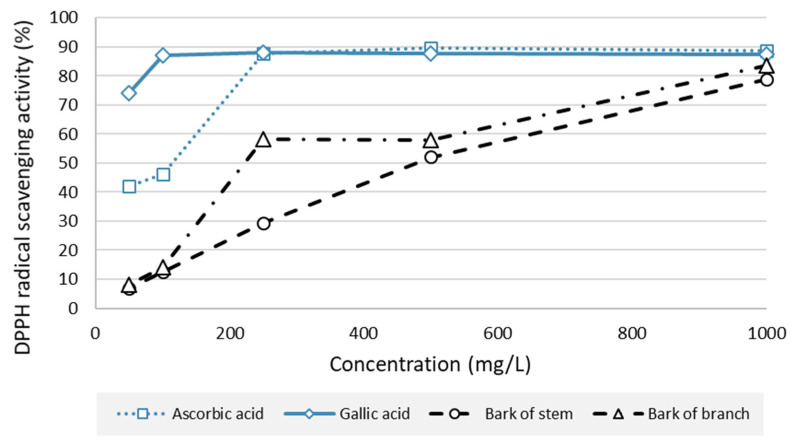
DPPH-radical-scavenging activity (RSA, %) of bark extracts of silver fir (*Abies alba*; bark of silver fir stem and bark of silver fir branch) and reference antioxidants (gallic acid, ascorbic acid) at different concentrations.

The results of HPLC analysis are given in Figure 3 and Table 3. The most abundant compounds in the stem bark sample of silver fir were catechin, epicatechin, taxifolin, ferulic acid and matairesinol (Figure 4). The identified compounds also included homovanillic acid, coumaric acid and secoisolariciresinol (Figure 3), while hydroxybenzoic acid, isolariciresinol, lariciresinol were found in the BSt extracts only in traces. We obtained two extracts from stem bark (BSt) and branch bark (BBr) with different chemical composition, both of which were relatively good scavengers of radicals (Figure 4 and Figure 5).

Both the HPLC trace of the prepared bark extract with a raised baseline (Figure 3) and the darkly coloured spot representing the place at which the bark sample was applied on the TLC plate (Table 2) can be explained by the presence of polar and high-molecular-weight extractives that were not sufficiently separated with the chromatographic methods used. This speculation needs more research attention and should be supported by using additional analytical tools, such as chromatography coupled with mass spectrometry.

Chemical analysis of the inner and outer bark revealed that the outer bark contained a higher content of phenolic extractives compared to the inner bark (Figure 2). Branch bark analysis revealed that the samples of branch bark taken near the stem contained a higher number of identified compounds compared to the samples taken from branches further away. We hypothesise that these data support the assumption that with a greater proportion of older/outer bark, the content of hydrophilic extractives, total phenolics (Figure 1 and Figure 2) and most individual compounds (Table 3) increases. Table 3 shows that the bark of the branches contained some compounds that were detected only in traces in the bark samples of the stems. These compounds were isolariciresinol, lariciresinol and secoisolariciresinol.

The antioxidative potential of silver fir bark extract (stem bark extract and branch bark extract) and control compounds (gallic acid and ascorbic acid) is presented in Figure 5.

The results of the DPPH test showed that the extracts from both stem bark (BSt) and branch bark (BBr) were relatively good radical scavengers (Figure 5). At a concentration of 1000 mg/L, both BSt and BBr extracts performed similarly to the references (Figure 5). At this concentration, an RSA of 78.75% was measured for BSt and 83.64% for BBr. Compared to the reference values, the RSA for BSt and BBr decreased by 39.30% and 32.32%, respectively, at a test concentration of 500 mg/L. At 200 mg/L, BBr proved to be a better radical scavenger than BSt (Figure 5). At the lowest test concentration, i.e., at and below 100%, BSt and BBr did not possess relevant radical-scavenging potential. As expected, gallic acid was confirmed as the most potent natural antioxidant in our study (Figure 5). The results of the DPPH test extend the previously published findings [12], i.e., they show the bark of branches to be a source of natural radical scavengers. In this context, a potential application of silver fir (*Abies alba*) bark as a bioactive agent in food supplements has also been suggested [12].

The results of our antifungal test showed that the bark extracts had no or relatively low antifungal effect towards the test organisms (Table 4). The growth inhibition of the test fungi was measured to be less than 12% in the best cases (Table 4). However, significant differences were found in the inhibition of fungi by the extracts (ANOVA, *p* < 0.050). We also found that the concentrated extracts from branch bark (BBr5 in Table 4) showed better inhibition of *S. commune*, *P. expynsum* and *F. solani* compared to the extracts from stem bark. The best inhibitory effect was shown by 5% bark extract towards *T. versicolor*. The extracts of stem bark and branch bark showed the best inhibitory effect on the growth of *P. expynsum* and the weakest effect on the growth of *S. commune* (Table 4). In comparison to the relatively strong antifungal effect measured, e.g., for pine knotwood extractives [21], the agar well diffusion method did not show water-soluble extractives of silver fir bark to be compounds with antifungal potential.

## 3. Materials and Methods

### 3.1. Chemicals

Methanol (HPLC grade), Folin–Ciocalteu phenol reagent (2 N), formic acid (≥99%) and anhydrous sodium carbonate (99%) were purchased from Sigma-Aldrich (Steinheim, Germany). Water and acetone, both HPLC grade, were purchased from J.T. Baker (Phillipsburg, NJ, USA). Cyclohexane (99%), ethyl acetate and dimethylsulphoxide (DMSO) were provided by Carlo Erba Reagents (Milano, Italy). Analytical standards used for the chromatographic analysis, secoisolariciresinol (purity (HPLC) ≥ 95%), pinoresinol (purity (HPLC) ≥ 95%), matairesinol (purity (HPLC) ≥ 95%) and quercetin (purity (HPLC) ≥ 95%) were obtained from Merck (Sigma-Aldrich Chemie, Taufkirchen, Germany). Epichatechin (purity (HPLC) ≥ 99%), coumaric acid (purity (HPLC) ≥ 90%), homovanillic acid (purity (HPLC) ≥ 95%), taxifolin (purity (HPLC) ≥ 99%) and ferulic acid (purity (HPLC) ≥ 90%) were purchased from Extrasynthese (Genay, France). Gallic acid monohydrate (HPLC assay, ≥99%), gallic acid (certified reference material), l-ascorbic acid (reagent grade), butylated hydroxyanisole (analytical reference material) and 2,2-diphenyl-1-picrylhydrazyl (DPPH) were provided by Merck (Sigma-Aldrich Chemie, Taufkirchen, Germany). Lariciresinol (purity ≥ 95%) and isolariciresinol (purity ≥ 95%) were kindly provided by our colleagues from the Laboratory of Organic Chemistry, Åbo Akademi University (Prof. Dr. Stefan Willför and Dr. Patrik Eklund). The potato dextrose agar (PDA) nutrient medium for fungal assay was purchased from Gram-Mol (Zagreb, Croatia) and DIFCO (Fisher Scientific, Franklin Lakes, NJ, USA). The fungal and mould isolates of *T. versicolor*, *S. commune*, *G. trabeum*, *P. expynsum* and *F. solani* originated from the fungal collection of the Biotechnical Faculty, University of Ljubljana.

### 3.2. Bark Material

The bark of three adult silver fir trees (*Abies alba* Mill.) was analysed. The trees were felled in mid-December 2018 in the forests of Kočevska Reka, Slovenia (45°34′31.5′′ N 14°46′27.8′′ E). Two stem discs were sawn from each felled tree at two different heights. The annual rings of the cross section were counted to determine the age of the disc. The disc diameter and bark thickness were measured for each of the sampled discs. Each measurement was performed twice, the second being taken in a perpendicular direction from the first one. All biometric data are collected in Table 5.

**Figure 6:**
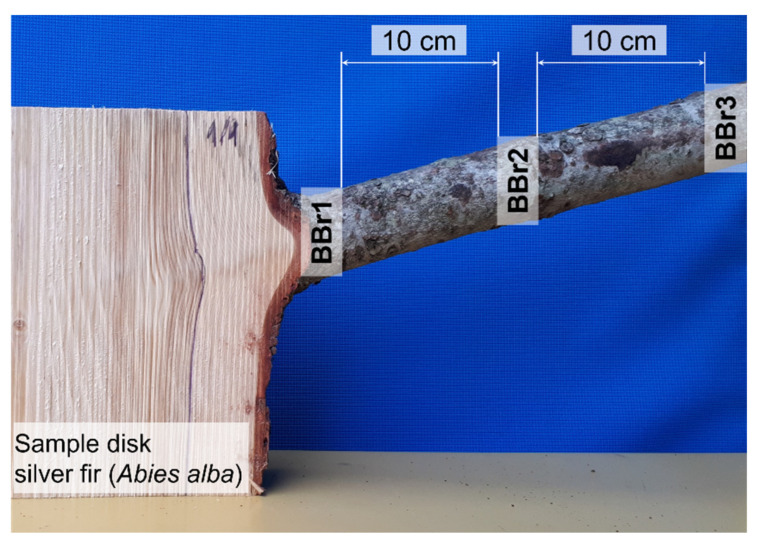
Branch bark samples removed along the branch of silver fir (*Abies alba*).

The bark of branches was also included in this investigation. The protocol of how the branch bark samples were taken is presented in Figure 6. Several bark samples were taken along the branch. The first sample of branch bark was removed at the point of the branch entering the stem (BBr1), the second sample (BBr2) was taken at a distance of 10 cm from the first one and the final and third branch bark sample (BBr3) was taken at a distance of 10 cm from the second sample.

After sampling, the collected material was oven-dried (SP 250-C, Kambič, Semič, Slovenia) at 40 °C for 24 h. The dried bark samples were then disintegrated on a Retsch SM 2000 cutting mill (Haan, Germany) using a 1 mm sieve. The disintegrated bark samples were stored in the dark at room temperature until further processing. Before extraction, the ground bark was overnight freeze-dried in a Telstar LyoQuest lyophilisator at 0.040 mbar and −85 °C for 24 h.

### 3.3. Moisture Content

The moisture content (*X_bark_*) of the bark was expressed as a ratio between the mass of water and the mass of fresh bark [22]. Fresh samples were weighed and placed in an oven (Kambič SP 250-C, Semič, Slovenia) at 103 ± 2 °C and then dried to constant weight. The bark moisture content was calculated using the following equation:

(1) $$X_{bark}=\frac{m_{b}-m_{a}}{m_{b}}\times 100,$$

where *m_b_* is the mass of fresh bark (g) and *m_a_* is the mass of dried bark (g).

### 3.4. Extraction of Bark

Bark extraction was carried out with accelerated solvent extraction in the Thermo Fisher Scientific Dionex system ASE 350 (Waltham, MA, USA). Briefly, 1 g of freeze-dried bark sample was weighed into a 10 mL SST extraction cell. Distilled water was used as the solvent for extraction. Extraction was carried out at 120 °C and 103.42 bar under a N_2_ atmosphere with 4 × 5 min static cycles. Extracts were filtered through a cellulosic filter, i.e., “on-line” during the extraction. The final bark extracts were prepared so that a sample-to-solvent ratio of 1:100 (*w/v*) was obtained. After the extraction, all the extracts were stored in amber-coloured glass bottles until further chemical analysis. 

### 3.5. Content of Hydrophilic Extractives

Hydrophilic extractives were measured gravimetrically. Empty test tubes were first dried in an oven at 103 ± 2 °C. Next, 10 mL of water extract was pipetted into each tube and placed into an oven at 103 ± 2 °C. The extracts were dried to constant weight. The contents of hydrophilic extractives in bark samples were expressed on a freeze-dried bark basis (mg/g dw).

### 3.6. Content of Total Phenols

Total phenols in the water extracts of bark were measured following a protocol already described [23,24]. Diluted Folin–Ciocalteu reagent and aqueous sodium carbonate solution (Na_2_CO_3_) were added to bark extracts. Gallic acid was used as the external standard for semi-quantitative analysis. The concentration range of the gallic acid solutions was from 1 mg/L to 500 mg/L, defining a linear calibration (*R*^2^ > 0.99). After 2 h of incubating the samples, absorbance was measured at a wavelength of 765 nm. Results were expressed in mass equivalents of gallic acid per dry bark basis (mg GAE/g dw).

### 3.7. Quality Check of Bark Extractives with Thin-Layer Chromatography

Thin-layer chromatography (TLC) was used as a simple and quick analytical tool for a quality check of the bark extracts, i.e., to find out how many different compounds and which compounds were present in the extracts. The bark extracts were completely dried and then dissolved in acetone. To separate the compounds of the extracts effectively, it was necessary to determine with a preliminary analysis which solvents to use as the mobile phase. Cyclohexane/ethyl acetate (60:40, *v/v*), cyclohexane/ethyl acetate (50:50, *v/v*), chloroform/ethyl acetate (50:50, *v/v*), methanol/chloroform (1:2, *v/v*), chloroform/ethanol (90:10, *v/v*) and dichloromethane/ethanol (93:7, *v/v*) were therefore tested. A mixture of chloroform and ethanol in a volume ratio of 90:10 was found to be the best solvent combination. The extracts were then separated on a silica gel TLC plate (silica Gel 60, F254 aluminium sheets, 20 cm × 20 cm; Merck, Darmstadt, Germany). The TLC plates were developed in a TLC chamber with a saturated atmosphere. The separate compounds were visualised with UV light and by spraying the plates with ferric chloride or a mixture of sulphuric acid and ethanol. Extractives were qualitatively evaluated on the basis of a compound retention time that was calculated following the equation:

(2) $$R_{f}=\frac{L_{S}}{L_{o}}$$

where *R_f_* is the retention factor, *L_S_* is the distance between the start line and the centre of a spot and *L_o_* is the distance between the start and the front of the mobile phase.

### 3.8. Analysis of Bark Extractives with High-Performance Liquid Chromatography

Detailed chemical analysis of individual compounds present in the bark extracts was performed with the Accela 600 system for high-performance liquid chromatography (HPLC; Thermo Fisher Scientific, Waltham, MA, USA). Extracts were first filtered through a 0.20 μm PA filter into amber-coloured 1.5 mL vials. The vials were inserted into a vial holder that was placed in the autosampler of the system. The autosampler was thermostated at 4 °C. Separation of the bark extractives was performed on a C18 chromatographic column (Accucore, 4.6 mm ID, length of 150 mm) filled with 2.6 µm stationary phase particles (Thermo Fisher Scientific, Waltham, MA, USA). Water (solvent A) and methanol (solvent b) were used as the mobile phase. Both solvents contained 0.1% of formic acid (*v/v*). The flow rate of the mobile phase was set at 1000 µL/min. Samples were measured in triplicate. Separated compounds were detected by means of a Thermo Fisher Scientific Accela photo diode array detector (PDA) characterised by a wavelength accuracy of ±1 nm at 254 nm and 640 nm (product specifications). Absorbance was measured at 280 nm, and UV spectra were recorded from 200 nm to 400 nm. The chemical identity of the separated compounds was determined by comparing the retention times and spectra of the separated compounds with the times and spectra of the analytical standards. The standards used for HPLC analysis are presented in Section 3.1. Linearity of the calibration curves was ensured, with *R*^2^ being >0.99. The results were calculated in milligrams of the compound identified per gram of freeze-dried bark (mg/g dw).

### 3.9. Antioxidant Potential of Bark Extractives

The antioxidant effect of the bark extractives of silver fir was measured using the 2,2-diphenyl-1-picrylhydrazyl (DPPH)-free-radical-scavenging method [13,25]. Briefly, the antioxidant properties of the bark extracts were compared to selected reference compounds, i.e., gallic acid (GA), ascorbic acid (AA) and butylhydroxyanisole (BHA). Water was used as a blank solution. The bark extracts of silver fir and the references were prepared as aqueous solutions in five concentrations (1000 mg/L, 500 mg/L, 250 mg/L, 100 mg/L and 50 mg/L). The solutions and the blank sample were placed into cuvettes, and an aqueous solution of DPPH reagent was added. The incubation of reaction mixtures lasted 30 min at room temperature. Absorbance was measured at a wavelength of 517 nm. The antioxidant potential of the samples was measured colorimetrically, whereby the DPPH-radical-scavenging activity (*RSA*) was calculated according to the equation:

(3) $$RSA\;\left[\%\right]=\left(\frac{A_{0}-A_{sample}}{A_{0}}\right)\times 100,$$

where *A*_0_ is the absorbance of a blank sample (water) and *A_sample_* is the absorbance of the sample or reference antioxidant.

### 3.10. In Vitro Antifungal Assay

**Figure 7:**
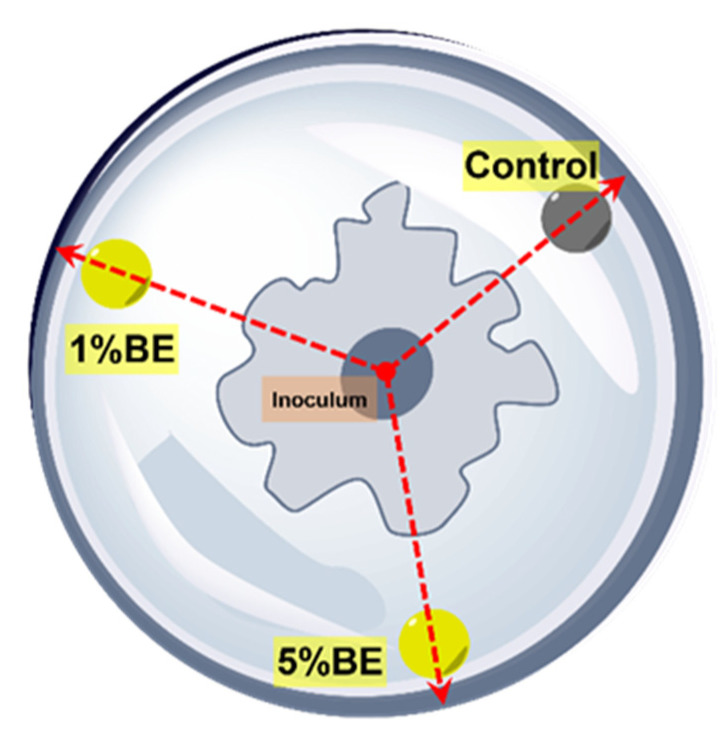
Schematic presentation of in vitro measurement of the inhibition effect of silver fir bark extracts towards wood-decaying fungi (*T. versicolor*, *S. commune* and *G. trabeum*) and moulds (*P. expynsum* and *F. solani*). The measurements on each Petri dish were always taken in a defined radial direction (red arrows).

The antifungal effect of the bark extracts of silver fir was evaluated in vitro by means of the agar well diffusion method [21,25]. Freeze-dried extracts of stem bark and branch bark were dissolved in dimethylsulphoxide (DMSO). Two test solutions for each extract were prepared, i.e., 1% and 5% (*w/v*, DMSO). The growth medium was prepared with potato dextrose agar (PDA). Three wells of 8 mm diameter were drilled in the media, with the centre of a well located 10 mm from the edge of the Petri dish, into which 100 µL of pure DMSO (blank sample), and 1% and 5% solution were pipetted. The test organisms selected were the fungi *Trametes versicolor* (Tv), *Schizophyllum commune* (Scc) and *Gloeophyllum trabeum* (Gt) and the moulds *Penicillium expynsum* (Pee) and *Fusarium solani* (Fus). The selected wood-decaying fungi and moulds are available in the culture collection of industrial microorganisms of the Biotechnical Faculty, University of Ljubljana [26]. Diffusion assay was started by placing inoculums and spore suspensions in the centre of the Petri dishes. Six replicates for each fungus or mould were prepared, and all the inoculated Petri dishes were placed in a growth chamber at 25 °C and 75% relative humidity. The growth of fungi was measured every 2/3 days for 3 weeks or until the organism grew in one direction to the edge of the Petri dish (Figure 7). The antifungal effect of the extracts is presented as the percentage inhibition of mycelium growth in a radial direction (*In*, %):

(4) $$In\;\left(\%\right)=\left(\frac{r_{in}}{r_{0}}\right)\times 100$$

where *r_in_* (mm) is the distance between the edge of the Petri dish and the mycelia and *r*_0_ (mm) is the distance between the edge of the Petri dish and the edge of the inoculum/spore suspension well. 

### 3.11. Statistical Analysis

The results were analysed for significant differences using basic statistical analysis with Statgraphics software. Analysis of variance (ANOVA) and Fisher’s least significant difference (LSD) procedure at a 95% confidence level were performed. The structures of the compounds were drawn using Perkin Elmer’s ChemDraw 20.1 software.

## 4. Conclusions

Our investigation showed that there were no significant differences in the content of water-soluble extractives between the stem bark and branch bark samples, although the stem bark contained the highest amounts of total phenols among the studied samples. High amounts of phenolic extractives were also measured in the branch bark samples taken from the base of the branch. Analysis of the separated tissues of silver fir stem bark indicated that the outer part of the stem bark is the richest source of phenolic extractives; significantly lower amounts of total phenols were measured in the inner parts of the bark. Qualitative chromatographic analysis with TLC and HPLC ascribed the most abundant signals to phenolic acids and flavonoids. Lignans were present in the extracts only in traces. The results of chromatography revealed that the extracts of silver fir bark contain large amounts of polar compounds that were not sufficiently separated and identified with the applied methods. Identifying and quantifying these compounds is one of the important goals of our future research activities. Extractives of both stem and branch bark were shown to be relatively strong free-radical scavengers, while a significant antifungal effect was not confirmed for the investigated extracts. The findings support the existing literature data, with the important information that in an adult silver fir, the tree bark of the branch bases and the outer parts of stem bark contain high amounts of phenolic extractives.

## Figures and Tables

**Table 1:** Moisture content of bark as a function of time.

	1st Sampling	2nd Sampling	3rd Sampling	4th Sampling	5th Sampling
	0 day	14 days	28 days	42 days	53 days
Moisture content (%)	42.526 (3.059)	40.751 (2.921)	37.753 (4.224)	36.644 (3.501)	35.235 (4.513)

**Table 2:** Compounds identified with thin-layer chromatography.

Thin-Layer Chromatogram	Unknown Compound	R_f_	Compound	TLC Spot HPLC Number
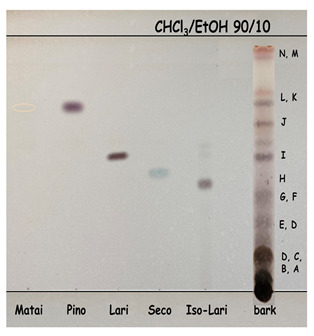	N	0.96	Isomers of pinoresinol + matairesinol	
	M	0.93		
	L	0.78	Matairesinol	10
	K	0.74	Pinoresinol	11
	J	0.66	Hydroxymatairesinol + allo–hydroxymatairesinol *	
	I	0.58	Lariciresinol	8
	H	0.53	Secoisolariciresinol	9
	G	0.39	Isolariciresinol	7
	F	0.35		
	E	0.27	Lignan A *	
	D	0.21	Oligolignans, * + polar compounds	
	C	0.16		
	B	0.13		
	A	0.05		

* Based on literature [20].

**Table 3:** Contents of identified phenolic compounds (mg/g, dw) in the bark of silver fir (*Abies alba*). Branch bark was sampled at various distances from the stem. BBr1, branch bark at the trunk; BBr2, branch bark 10 cm away from the stem; BBr3, branch bark 20 cm away from the stem.

Bark Sample *	Catechin (mg/g)	Epicatechin (mg/g)	Taxifolin (mg/g)	Ferulic Acid (mg/g)	Matairesinol (mg/g)	Hydroxybenzoic Acid (mg/g)	Homovanillic Acid (mg/g)	Coumaric Acid (mg/g)	Isolariciresinol (mg/g)	Lariciresinol (mg/g)	Secoisolariciresinol (mg/g)
BBr1	0.755	0.782	0.291	0.027	0.201	0.000	0.000	0.104	0.094	0.035	0.042
BBr2	0.719	0.809	0.342	0.036	0.105	0.013	0.000	0.079	0.019	0.000	0.016
BBr3	0.683	0.664	0.378	0.024	0.138	0.012	0.025	0.088	0.022	0.000	0.031

* The location of branch bark samples is presented in Figure 6.

**Table 4:** Inhibition of white-rot- and brown-rot-producing fungi and moulds by bark extracts of silver fir (*Abies alba*) *.

Extract		*Trametes versicolor* (%In)		*Schizophyllum* *commune* (%In)		*Gloeophyllum trabeum* (%In)		*Penicillium expynsum* (%In)		*Fusarium oxysporum* (%In)
**C**		0.00 ± 0.00 ^a^		0.00 ± 0.00 ^a^		0.00 ± 0.00 ^a^		0.00 ± 0.00 ^a^		0.00 ± 0.00 ^a^
**BSt1**		6.61 ± 3.62 ^a.b^		3.43 ± 4.31 ^a.b.c^		3.25 ± 2.85 ^a^		2.35 ± 3.85 ^a.b^		1.07 ± 0.99 ^a.b^
**BSt5**		9.26 ± 7.40 ^b^		4.15 ± 2.60 ^b.c^		8.47 ± 4.20 ^b^		1.86 ± 2.58 ^a.b^		0.00 ± 0.00 ^a^
**BBr1**		4.93 ± 5.71 ^a.b^		1.32 ± 2.94 ^a.b^		0.00 ± 0.00 ^a^		5.60 ± 3.92 ^b.c^		2.90 ± 1.83 ^c^
**BBr5**		9.60 ± 9.81 ^b^		7.52 ± 5.75 ^c^		11.16 ± 5.39 ^b^		8.82 ± 6.54 ^c^		2.59 ± 0.94 ^b.c^

* Results are expressed as the mean value of measurements with standard deviations. The different letters a–c within the same column indicate statistically significant differences at a 95.0% confidence level (Fisher’s least significant difference procedure). Water extracts of silver fir (*Abies alba*): C, control; BSt1 and BSt2, extracts of stem bark (1% and 5%, *w/v*); BBr1 and BBr2, extracts of branch bark (1% and 5%, *w/v*).

**Table 5:** Biometric data of the sample trees of silver fir (*Abies alba*).

Tree	Stem Disc	Age (Number of Annual Rings)	Height of Disc Sampling (m)	Diameter of Disc (cm)	Bark Thickness (cm)
1	1	64	12	28.90	1.05
	2	55	16	23.75	0.75
2	1	61	7	39.15	1.85
	2	39	16	28.10	0.8
3	1	62	13	40.70	1.55
	2	31	21	25.75	1.7

## Data Availability

The data presented in this study are available on request from the corresponding authors.

## Sample Availability

Not available.
